# Supplementary material for: An Alternative Binding Mode of IGHV3-53 Antibodies to the SARS-CoV-2 Receptor Binding Domain
Source: Cell Rep. 2020 Sep 29;33(3):108274. doi: 10.1016/j.celrep.2020.108274 (PMC7522650; doi:10.1016/j.celrep.2020.108274)
Supplement: Document S1. Figures S1–S5 and Tables S1 and S2 [file mmc1.pdf]

Cell Reports, Volume *33*

Supplemental Information

An Alternative Binding Mode of IGHV3-53 Antibodies to the SARS-CoV-2 Receptor Binding Domain

Nicholas C. Wu, Meng Yuan, Hejun Liu, Chang-Chun D. Lee, Xueyong Zhu, Sandhya Bangaru, Jonathan L. Torres, Tom G. Caniels, Philip J.M. Brouwer, Marit J. van Gils, Rogier W. Sanders, Andrew B. Ward, and Ian A. Wilson

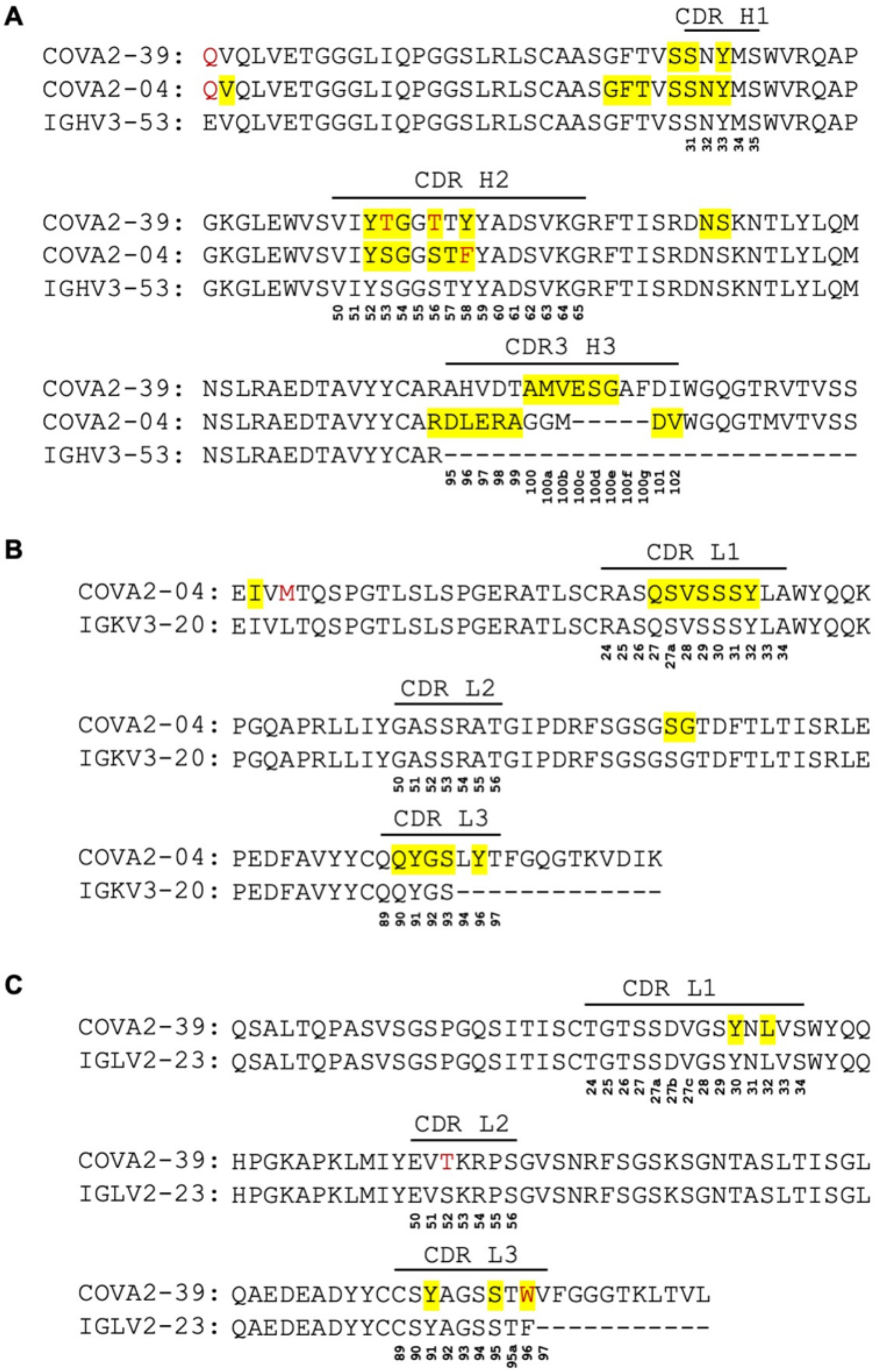

Figure S1, related to Figure 1. Comparison of COVA2-04 and COVA2-39 sequences to germline sequences. (A) Alignment of the heavy-chain variable domain sequences of

COVA2-04 and COVA2-39 with the germline IGHV3-53 sequence **(B)** Alignment of the light-chain variable domain sequence of COVA2-04 with the germline IGKV3-20 sequence. **(C)** Alignment of the light-chain variable domain sequence of COVA2-39 with the germline IGLV2-23 sequence. The regions that correspond to CDR H1, H2, H3, L1, L2, and L3 are indicated. Residues that differ from the germline are highlighted in red. Residue positions in the CDRs are labeled according to the Kabat numbering scheme. Residues that interact with the RBD are highlighted in yellow.

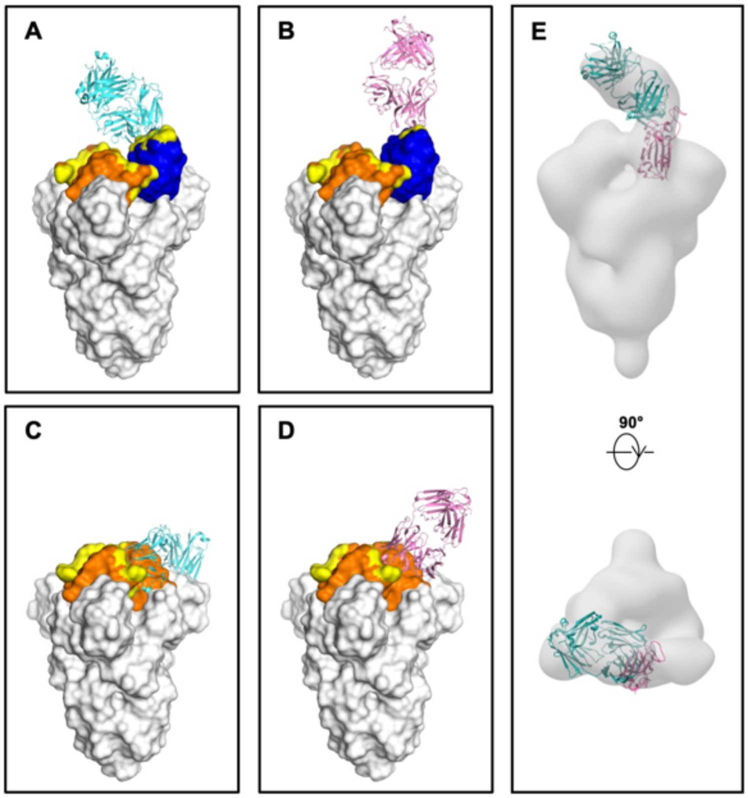

Figure S2, related to Figures 1 and 2. Modelling the binding of COVA2-39 and COVA2-04 on the homotrimeric SARS CoV-2 spike (S) protein. (A-B) The SARS-CoV-2 S trimer is shown with one RBD in the up conformation (cyan) and two RBDs in the down conformation (orange) (PDB: 6VSB) (Wrapp et al., 2020)**.** Binding of **(A)** COVA2-04 (cyan) and **(B)** COVA2-39 (pink) to the RBDs in the up conformations is modelled. **(C-D)** The SARS-CoV-2 S trimer is shown with all three RBD in down conformations (PDB 6VXX) (Walls et al., 2020). **(C)** COVA2-04 (cyan) and **(D)** The epitope for COVA2-39 (pink) is partially buried in the RBD down conformation that suggests that it is fully accessible for binding only in the up conformation. Epitopes are colored in yellow. **(E)** COVA2-04/RBD crystal structure was fitted here to the negative-stain electron microscopy (nsEM) reconstruction of the Fab COVA-2-04/SARS CoV-2 spike protein complex that was previously generated (Brouwer et al., 2020). A similar analysis was not performed with the

COVA2-39/RBD because of the poorer quality of nsEM map of the COVA2-39/S protein complex (Brouwer et al., 2020).

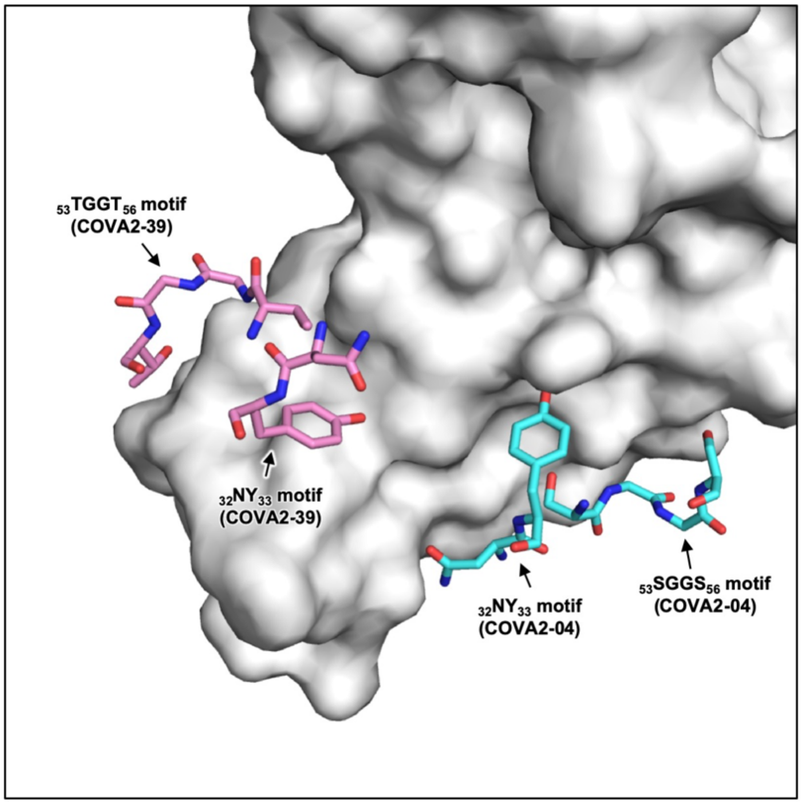

Figure S3, related to Figure 3. Locations of ${}_{32}$NY${}_{33}$ motif and ${}_{53}$SGGS${}_{56}$ (${}_{53}$TGGT${}_{56}$) motifs when VH3-53 antibodies bind the SARS CoV-2 RBD. The locations of ${}_{32}$NY${}_{33}$ motif and ${}_{53}$SGGS${}_{56}$ motif in COVA2-04 (cyan) as well as ${}_{32}$NY${}_{33}$ motif and ${}_{53}$TGGT${}_{56}$ motif in COVA2-39 (pink) are shown. SARS-CoV-2 RBD is shown as a white surface.

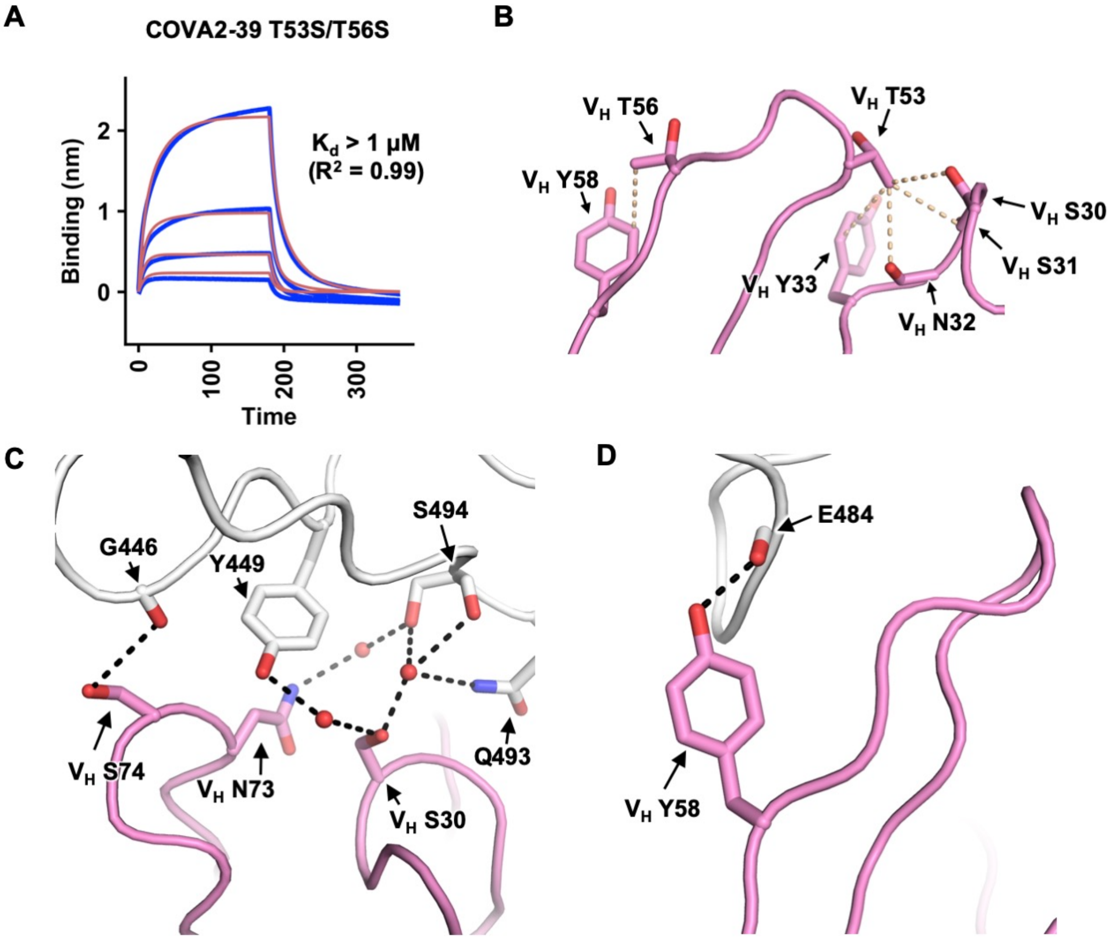

Figure S4, related to Figure 3. Key interactions between COVA2-39 and RBD. (A) Binding kinetics of COVA2-39 T53S/T56S somatic revertant in Fab format to SARS-CoV-2 RBD were measured by biolayer interferometry (BLI). Y-axis represents the response and blue lines represent the response curves. A 1:1 binding model did not fit very well, potentially due to some contribution of non-specific binding to the response curve. Subsequently, a 2:1 heterogeneous ligand model was used to improve the fit, which is represented by the red lines. In both models, the K_d estimated is > 1 μM. Binding kinetics were measured for four concentrations of each Fab at 2-fold dilution starting from 500 nM. The K_d and R^2 of the fitting are indicated. Representative result of two replicates is shown here. **(B)** Van der Waals Interactions (within a distance of 4 Å, wheat dashed lines) that involve the methyl group of V_H T53 and V_H T56 of COVA2-39 are shown. **(C)** The hydroxyl

side chain of V_H S30 interacts with RBD Y449, Q493, and S494 through water-mediated H-bonds. The side chain of V_H N73 interacts with RBD S494 through a water-mediated H-bond. The side chain of V_H S74 H-bonds with the backbone carbonyl of RBD G446. **(D)** The side chain of V_H Y58 H-bonds with the backbone carbonyl of RBD E484. Hydrogen bonds are represented by dashed lines and water molecules by red spheres.

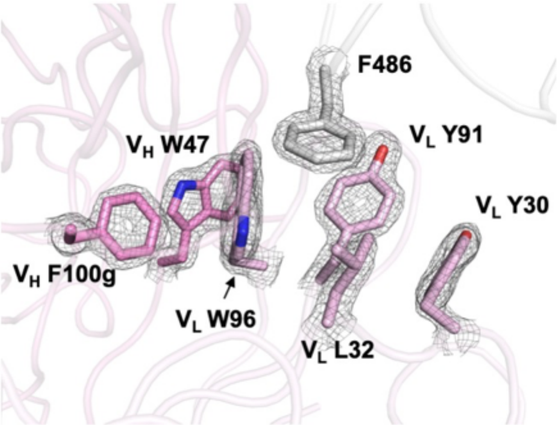

Figure S5, related to Figure 5. Electron density maps for the ridge-anchoring pocket. Final 2Fo-Fc electron density map for the ridge-anchoring pocket around RBD F486 is contoured at 2.0 σ.

Table S1, related to Figure 1. X-ray data collection and refinement statistics

**Data collection**		
	COVA2-04 + RBD	COVA2-39 + RBD
Beamline	SSRL 12-1	SSRL 12-1
Wavelength (Å)	0.97946	0.97946
Space group	C 1 2 1	P 1 2_1 1
Unit cell parameters		
a, b, c (Å)	197.2, 84.7, 57.3	68.9, 80.4, 72.0
α, β, γ (°)	90, 99.6, 90	90, 104.9, 90
Resolution (Å) [a]	50.0–2.35 (2.39–2.35)	50.0–1.72 (1.75–1.72)
Unique reflections [a]	37,264 (3,371)	79,114 (7,573)
Redundancy [a]	2.9 (2.0)	1.9 (1.8)
Completeness (%) [a]	97.2 (88.6)	97.2 (97.6)
$<I/\sigma_I>$ [a]	5.4 (1.1)	26.2 (1.1)
R_{sym} [b] (%) [a]	16.3 (89.8)	5.7 (93.4)
R_{pim} [b] (%) [a]	7.2 (47.0)	3.2 (57.6)
$CC_{1/2}$ [c] (%) [a]	98.5 (67.7)	99.6 (50.6)
**Refinement statistics**		
Resolution (Å)	48.6–2.35	34.8–1.72
Reflections (work)	37,264	79,095
Reflections (test)	3,371	7,569
R_{cryst} [d] / R_{free} [e] (%)	19.7/23.8	17.8/21.1
No. of atoms	4,903	5,350
Macromolecules	4,765	4,775
Glycans	28	14
Solvent	124	561
Average *B*-value ($Å^2$)	52	38
Macromolecules	52	37
Glycans	30	69
Solvent	49	41
Wilson *B*-value ($Å^2$)	45	26
**RMSD from ideal geometry**		
Bond length (Å)	0.007	0.018
Bond angle (°)	1.15	1.47
**Ramachandran statistics (%)**		
Favored	97.1	97.9
Outliers	0.16	0.00
**PDB code**	7JMO	7JMP

[a] Numbers in parentheses refer to the highest resolution shell.
[b] $R_{sym} = \Sigma_{hkl} \Sigma_i | I_{hkl,i} - <I_{hkl}> | / \Sigma_{hkl} \Sigma_i I_{hkl,i}$ and $R_{pim} = \Sigma_{hkl} (1/(n-1))^{1/2} \Sigma_i | I_{hkl,i} - <I_{hkl}> | / \Sigma_{hkl} \Sigma_i I_{hkl,i}$, where $I_{hkl,i}$ is the scaled intensity of the ith measurement of reflection h, k, l, $<I_{hkl}>$ is the average intensity for that reflection, and *n* is the redundancy.
[c] $CC_{1/2}$ = Pearson correlation coefficient between two random half datasets.
[d] $R_{cryst} = \Sigma_{hkl} | F_o - F_c | / \Sigma_{hkl} | F_o | \times 100$, where F_o and F_c are the observed and calculated structure factors, respectively.
[e] R_{free} was calculated as for R_{cryst}, but on a test set comprising 5% of the data excluded from refinement.

Table S2, related to Figure 2. Hydrogen bonds and salt bridges identified at the antibody-RBD interface using the PISA program.

**COVA2-04**	**Distance [Å]**	**SARS-CoV-2 RBD**
	Hydrogen bonds	
H:THR28[N]	3.1	A:ALA475[O]
H:ASN32[ND2]	3.1	A:ALA475[O]
H:TYR33[OH]	2.6	A:LEU455[O]
H:SER53[N]	3.5	A:TYR421[OH]
H:SER53[OG]	3.1	A:TYR421[OH]
H:SER53[OG]	2.4	A:ARG457[O]
H:GLY54[N]	2.9	A:TYR421[OH]
H:SER56[OG]	2.3	A:ASP420[OD2]
H:ARG94[NH1]	3.1	A:ASN487[OD1]
H:ARG94[NH2]	3.2	A:ASN487[OD1]
H:ARG94[NH2]	3.7	A:TYR489[OH]
H:ARG98[NE]	3.9	A:GLN493[OE1]
H:ARG98[NH1]	3.1	A:SER494[O]
H:GLY26[O]	2.6	A:ASN487[ND2]
H:SER31[O]	2.7	A:TYR473[OH]
H:GLU97[OE1]	3.2	A:LYS417[NZ]
H:GLU97[OE2]	2.8	A:TYR453[OH]
L:TYR32[OH]	3.5	A:TYR453[OH]
L:SER31[OG]	3.6	A:TYR495[O]
L:SER29[OG]	3.8	A:GLY496[O]
L:SER27A[OG]	3.2	A:THR500[O]
L:SER29[OG]	3.7	A:ASN501[OD1]
L:SER93[OG]	2.7	A:TYR505[OH]
L:SER93[N]	3.8	A:TYR505[OH]
L:GLY92[O]	2.9	A:ARG403[NH1]
L:SER93[OG]	3.8	A:ARG403[NH1]
	Salt bridges	
H:GLU97[OE1]	3.2	A:LYS417[NZ]

**COVA2-39**	**Distance [Å]**	**SARS-CoV-2 RBD**
	Hydrogen bonds	
H:THR53[N]	3.4	A:GLU484[OE2]
H:THR53[OG1]	3.6	A:GLU484[OE2]
H:GLY54[N]	2.8	A:GLU484[OE2]
H:THR56[N]	3.3	A:GLU484[OE1]
H:THR56[OG1]	2.7	A:GLU484[OE1]
H:TYR58[OH]	2.6	A:GLU484[O]
H:GLU100C[N]	3.8	A:ASN487[OD1]
H:GLU100C[N]	2.9	A:TYR489[OH]
H:GLU100C[OE2]	3.0	A:ASN487[N]
H:GLU100C[OE2]	2.5	A:TYR489[OH]
H:SER30[O]	3.2	A:GLN493[NE2]
L:SER95[OG]	2.8	A:PHE486[N]
